# DNA methylation changes in endometrium and correlation with gene expression during the transition from pre-receptive to receptive phase

**DOI:** 10.1038/s41598-017-03682-0

**Published:** 2017-06-20

**Authors:** Viktorija Kukushkina, Vijayachitra Modhukur, Marina Suhorutšenko, Maire Peters, Reedik Mägi, Nilufer Rahmioglu, Agne Velthut-Meikas, Signe Altmäe, Francisco J. Esteban, Jaak Vilo, Krina Zondervan, Andres Salumets, Triin Laisk-Podar

**Affiliations:** 1 Competence Centre on Health Technologies, Tartu, Estonia; 2 Institute of Molecular and Cell Biology, University of Tartu, Tartu, Estonia; 3 Estonian Genome Center, University of Tartu, Tartu, Estonia; 4 Institute of Computer Science, University of Tartu, Tartu, Estonia; 5 Women’s Clinic, Institute of Clinical Medicine, University of Tartu, Tartu, Estonia; 6 Wellcome Trust Centre for Human Genetics, University of Oxford, Oxford, UK; 7 Department of Women’s and Children’s Health, Division of Obstetrics and Gynecology, Karolinska Institutet, Stockholm, Sweden; 8 Department of Experimental Biology, University of Jaén, Jaén, Spain; 9 Endometriosis CaRe Centre, Nuffield Department of Obstetrics & Gynaecology, John Radcliffe Hospital, University of Oxford, Oxford, UK; 10 Institute of Bio- and Translational Medicine, University of Tartu, Tartu, Estonia; 11 Department of Obstetrics and Gynecology, University of Helsinki and Helsinki University Hospital, Helsinki, Finland

## Abstract

The inner uterine lining (endometrium) is a unique tissue going through remarkable changes each menstrual cycle. Endometrium has its characteristic DNA methylation profile, although not much is known about the endometrial methylome changes throughout the menstrual cycle. The impact of methylome changes on gene expression and thereby on the function of the tissue, including establishing receptivity to implanting embryo, is also unclear. Therefore, this study used genome-wide technologies to characterize the methylome and the correlation between DNA methylation and gene expression in endometrial biopsies collected from 17 healthy fertile-aged women from pre-receptive and receptive phase within one menstrual cycle. Our study showed that the overall methylome remains relatively stable during this stage of the menstrual cycle, with small-scale changes affecting 5% of the studied CpG sites (22,272 out of studied 437,022 CpGs, FDR < 0.05). Of differentially methylated CpG sites with the largest absolute changes in methylation level, approximately 30% correlated with gene expression measured by RNA sequencing, with negative correlations being more common in 5′ UTR and positive correlations in the gene ‘Body’ region. According to our results, extracellular matrix organization and immune response are the pathways most affected by methylation changes during the transition from pre-receptive to receptive phase.

## Introduction

DNA methylation is a type of epigenetic modification of post-replicative DNA, where a methyl residue is covalently added to the cytosine nucleotides. This dynamic process is catalysed by DNA methyltransferases and is essential for all mammalian cells. It has been shown that human tissues have each its own specific methylation pattern which contributes to tissue-specific transcription pattern and thereby to tissue development and specific functions^1^. The uterine inner lining, the endometrium, is a unique tissue because it undergoes histologically and functionally distinguished cyclic phases of growth and atrophy under the control of ovarian steroid hormones estrogen and progesterone. The proper functioning of the endometrium is needed to support the implantation of the embryo in the mid-secretory phase of the menstrual cycle. Endometrial receptivity or ‘window of implantation’ (WOI) has some inter-individual variation in the timing but occurs approximately a week after ovulation on cycle days 19–24.

Transcriptome studies have demonstrated a myriad of changes in endometrial gene expression during the transition from pre-receptive to receptive phase^2, 3^, and a specific transcriptome signature has been detected that is now used to determine the individual WOI and aid in selecting the best day for embryo transfer in women undergoing *in vitro* fertilization^4^. Although the endometrial function is believed to be under epigenetic control^5^, less is known about how endometrial DNA methylation pattern changes throughout the menstrual cycle, what impact it has on gene expression, and whether aberrations in methylation pattern could lead to altered endometrial function. According to recent studies, the endometrial methylome might indeed be dynamic throughout the menstrual cycle^6, 7^, correlate with changes in the transcriptome^6, 7^ and also play a role in the pathogenesis of endometrial disorders by affecting the expression of genes relevant for maintaining proper endometrial function^6, 8–10^. However, none of the previous studies have used genome-wide technologies to target directly the establishment of endometrial receptivity, therefore, we lack an understanding on how global DNA methylation changes and concomitant changes in gene expression occurring in a limited time-frame could contribute to controlling endometrial receptivity.

In order to better understand how DNA methylation changes might modify endometrial receptivity or the susceptibility to endometrial pathologies, we need a more thorough understanding on the normal endometrial methylome that corresponds to the restructuring of the endometrial tissue. We hypothesized that the transcriptomic changes observed in endometrial tissue around the time of embryo implantation are at least partially caused by changes in global DNA methylation pattern. Therefore, the aim of the present study was to use genome-wide technologies to characterize the endometrial methylome in pre-receptive and receptive endometrium sampled from the same individual within the same menstrual cycle. To find differentially methylated sites with higher confidence and obtain more robust results, we used a combination of three analysis methods, and to evaluate the potential effect of DNA methylation on gene expression, we tested for correlation between DNA methylation and gene expression levels. Finally, pathway analysis was used to put the findings into a wider biological context.

## Results

### General profiling

We studied the genome-wide DNA methylation profiles in endometrial biopsies from two time-points, pre-receptive (LH + 2) and receptive (LH + 8), in one menstrual cycle from 17 healthy, fertile-aged women. Of the 437,022 CpGs remaining for analysis after quality control, 19% (83,728) were consistently hypermethylated (β > 0.8), while 33% (145,385) were hypomethylated (β < 0.2) in both pre-receptive and receptive time-points.

**Figure 1:**
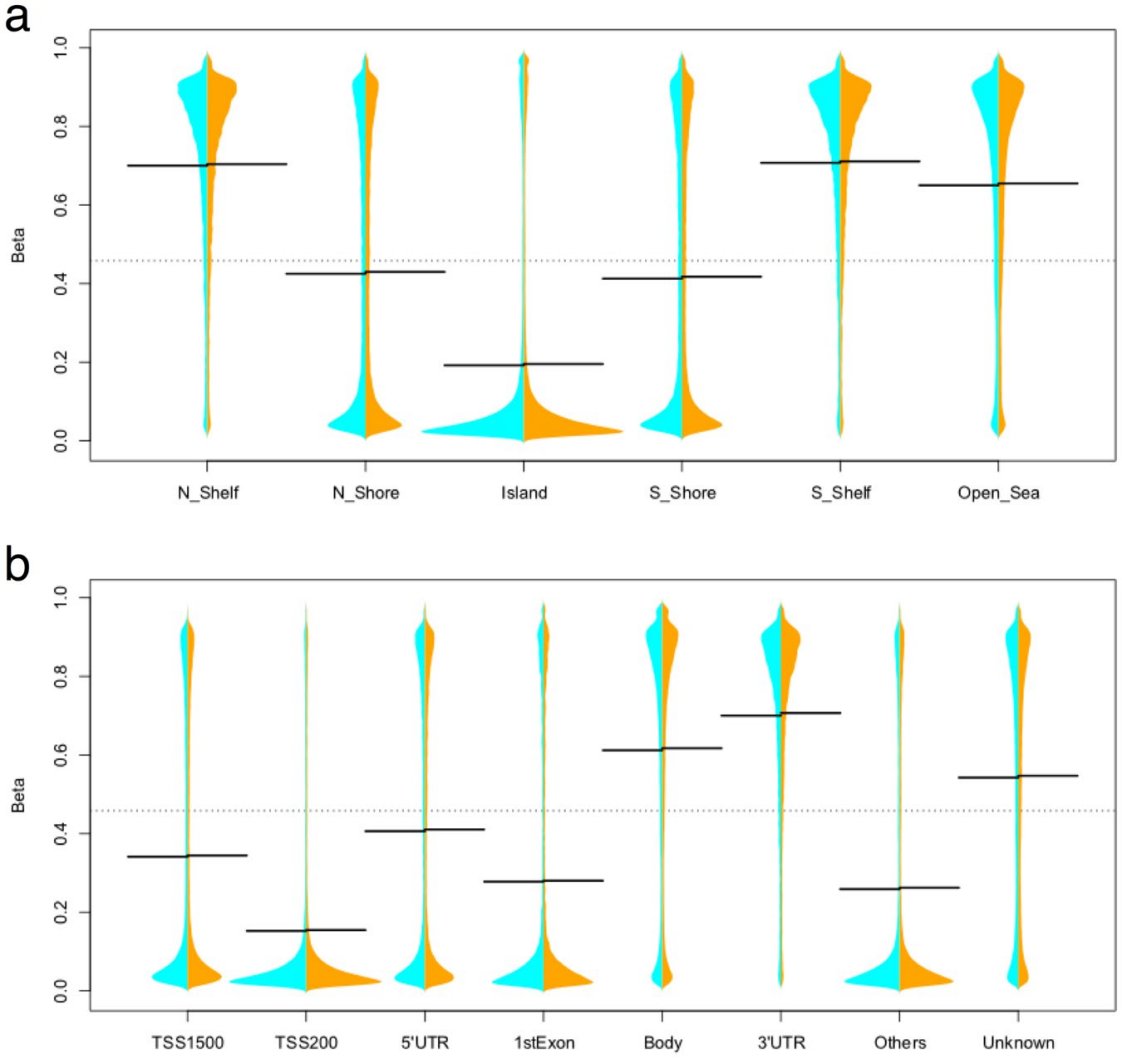
Methylation levels in pre-receptive (cyan, left) and receptive (orange, right) endometrium represented as split beanplots. The width of the plot represents the distribution of data, the black line shows the mean methylation value in group, while the dashed black line represents the overall average methylation level. (**a**) According to location (relative to CpG islands). The x-axis denotes the CpG island location while the y-axis denotes methylation β-values (0 to 1). (**b**) According to the region functional categories. The x-axis denotes the functional group while the y-axis denotes methylation β-values (0 to 1). CpGs annotated to multiple gene locations are labelled as ‘Others’, and CpGs with unknown annotations are labelled as ‘Unknown’.

**Figure 2:**
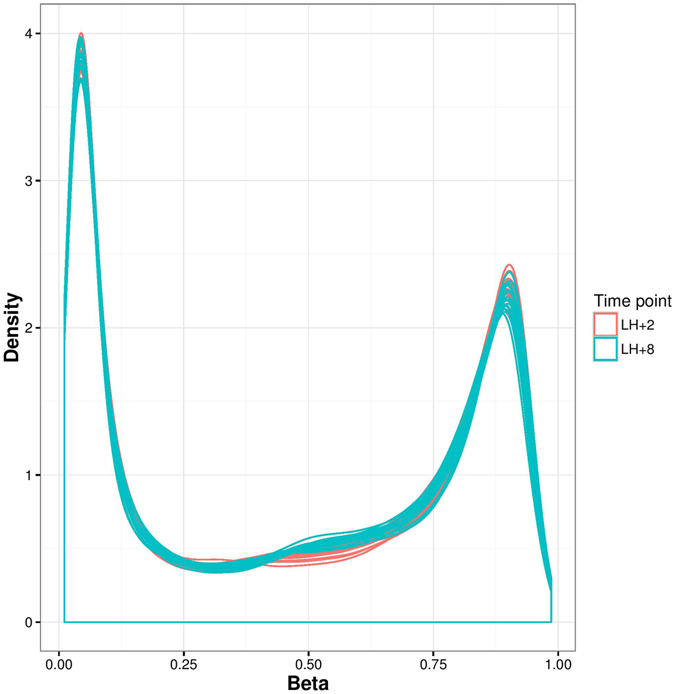
Density plot of DNA methylation levels (as β values) for pre-receptive (LH + 2) and receptive (LH + 8) endometrium samples from 17 women.

To test for differences in methylation value distributions between genomic regions, we carried out pairwise comparisons using the Kolmogorov-Smirnov test (for all comparisons presented here, p < 2.2 × 10^−16^). With regards to genomic location, CpG sites in CpG islands (CGIs) showed relatively lower methylation levels than CpG sites located in shelves (regions spanning 2–4 kb up- and downstream of the CpG islands), whereas the methylation levels of sites in CpG shores (regions spanning <2 kb up- and downstream of the CpG islands) followed a more uniform distribution, both in pre-receptive and receptive time-points (Fig. 1a). CpG sites in TSS1500 (−200 to −1,500 bases upstream of the transcription start site, TSS) showed slightly higher methylation levels compared to TSS200 (up to −200 bases upstream of TSS) regions of the gene promoters (Fig. 1b). On average, promoter regions exhibited lower methylation levels than gene body regions, supporting the claim that genomic regions involved in active transcription are hypomethylated resulting in accessibility to transcription factors^1^. Overall, the methylation profiles of samples from pre-receptive and receptive endometrium were relatively similar, with no great-magnitude changes (Fig. 2).

### Differential methylation

**Figure 3:**
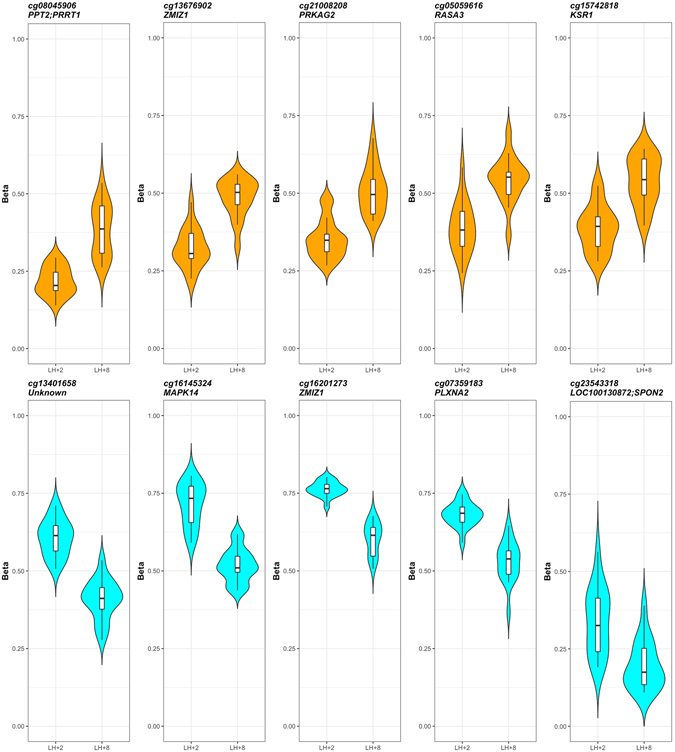
CpG-level differential methylation analysis results. Methylation levels of top 10 CpG sites differentially methylated between pre-receptive and receptive endometrium. Each plot represents a single CpG site and the gene it was annotated to. Upper panel (orange) – higher methylation in receptive endometrium; lower panel (light blue) – lower methylation in receptive endometrium.

For differential methylation analysis, we used a combination of three different methods to increase the possibility of identifying true positive results. Single CpG-level analysis resulted in 53,371 (12.2% of total) differentially methylated CpGs using RnBeads, 28,994 (6.6%) using Wilcoxon’s signed rank test and 55,086 (12.6%) using seqlm (all analyses were adjusted for age). The intersect of the three analysis methods resulted in 22,272 CpGs (5.1%) associated with 5,979 genes as differentially methylated between pre-receptive and receptive endometrium (Supplementary Figure 2) and were considered as the most likely set of truly differentially methylated CpGs (Supplementary Table 1). The same set of CpGs was used in all further single CpG site-level analyses. Changes in methylation levels included both increased (n = 18,820 CpG sites; 4.3% of all CpGs; 84.5% from differentially methylated CpGs; delta-β mean = 0.059, median = 0.057) and decreased (n = 3,452 CpG sites, 0.8% of all CpGs, 15.5% of differentially methylated CpGs; delta-β mean = −0.052, median = −0.051) methylation in receptive phase samples. A total of 842 CpG sites had a delta-β absolute value more than 0.1. The top ten sites with the largest methylation differences between pre-receptive and receptive endometrium are shown on Fig. 3. Clustering analysis using the 22,272 differentially methylated CpGs resulted in two main branches that divided the analysed samples according to menstrual cycle phase (pre-receptive and receptive). The first branch included all pre-receptive phase samples, except for one which clustered together with receptive phase samples. In addition, three receptive phase samples also clustered in the first branch (Supplementary Figure 3).

The region level analysis of all CpGs revealed 2,026 significant differentially methylated regions (DMRs; defined as at least 3 differentially methylated CpGs within a 500 bp window) (False Discovery Rate adjusted p-value, FDR < 0.05; Supplementary Table 2), of which 1,650 exhibited increased (associated with 1,217 genes) and 376 decreased (associated with 276 genes) methylation in receptive phase samples. 48 genes were present in both lists, depending on the location of the DMR. The most significant DMRs included CpGs in the ‘Open Sea’ region ~31 kb downstream from *IGF2*, in the ‘Body’ region of *PDLIM2* and the 3′ UTR region of *ZMIZ1*. *ZMIZ1* was also one of the genes highlighted in site-level analysis (Fig. 3).

**Figure 4:**
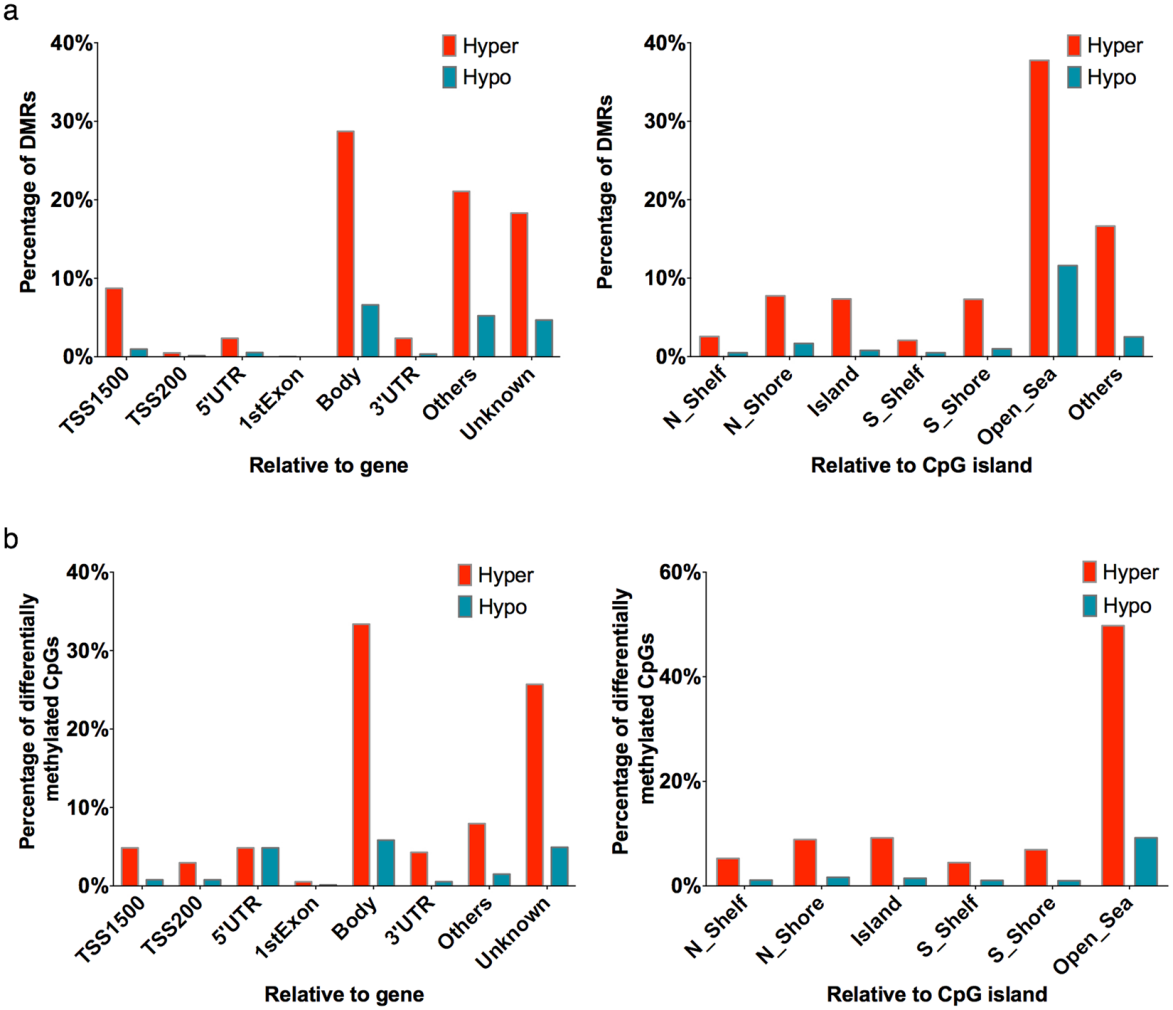
Location of differentially methylated sites and regions in relation to functional subregions and CpG islands. (**a**) Region-level analysis. (**b**) CpG-level analysis.

We also examined the location of differentially methylated CpG sites and regions in relation to gene sub-regions (TSS200, TSS1500, 5′ UTR, 1^st^ Exon, Gene body, 3′ UTR) and CpG islands (N_Shelf, N_shore, CpG island, S_Shelf, S_Shore, remaining sequences termed as ‘Open Sea’). Figure 4a and b represent the distribution of DMRs and differentially methylated CpGs. It can be clearly seen that gene body region exhibits highest differential methylation in both region and site level analyses. However, differential methylation mapped to multiple locations (represented as ‘Others’) was more common (up to 21% for DMRs related to increased methylation in receptive phase) in region level analysis than the site level analysis. This could be owing to the fact that methylation levels of nearby CpGs from multiple locations were spatially correlated and grouped into a single DMR. Large proportion of these differentially methylated regions/sites could not be annotated to known gene sub-regions (shown as ‘Unknown’) and only a negligible portion of them were located in promoter (TSS200 and TSS1500) and other genomic regions (5′ UTR, 3′ UTR and 1^st^ Exon). Regarding localization relative to CpG island, majority (up to 60%) of differentially methylated regions/sites were located in ‘Open Sea’. Comparing to the overall distribution of all analysed sites (n = 437,022), the distribution of differentially methylated CpG sites was significantly different for both in relation to gene-subregions and CGIs ($\chi$ ^2^ p-value for both < 2.2 × 10^−16^). This was characterized by under-representation in CGIs (10.7% of significant *vs*. 31.6% of all CpGs) and TSSs (9.5% of significant *vs*. 21.1% of all CpGs), and over-representation in ‘Open Sea’ (59.0% of significant *vs*. 35.4% of all CpGs), gene body (39.2% of significant *vs*. 31.0% of all CpGs) and ‘Unknown’ (30.6% of significant *vs*. 23.3% of all CpGs) regions.

### Correlation between methylation and gene expression

To characterize the potential effect of methylation status on gene expression levels, we used RNA sequencing data to evaluate the expression change of differentially methylated genes in the same samples. For the correlation analysis, only significantly differentially methylated CpG sites with an absolute delta-β value > 0.1 were used. In addition, we used only Illumina annotated CpGs and transcript pairs, which excluded all CpGs in ‘Open sea’ and resulted in 464 genes and 531 CpGs in total for analysis (altogether 546 pairs, as some CpGs were annotated to more than one gene). Correlation analysis showed 169 significantly correlated gene-CpG site pairs [that is 157 (34%) of tested genes and 168 (32%) of tested sites] (permutation p-value < 0.05) (Supplementary Table 3). Overall, the average proportion of significantly correlated CpGs was around 30%, but showed significant variation across different regions ranging from 22% in the 1^st^ Exon to 38% in the 5′ UTR (Table 1). The proportion of positive and negative correlations also varied in different regions, negative correlations being more common in the 5′ UTR and 1^st^ Exon, while positive correlations were more prevalent in the Body region (Table 1), consistent with the ‘DNA methylation paradox’^11^.

**Table 1:** Correlations between CpG site methylation and gene expression.

Region	Differentially methylated CpGs in region (n)	CpGs correlated with gene expression n (%)	Positively correlated CpGs n (%)	Negatively correlated CpGs n (%)
5′ region	145	45 (31.0%)	20 (44.4%)	25 (55.6%)
1st exon	18	4 (22.2%)	1 (25.0%)	3 (75.0%)
TSS200	16	4 (25.0%)	2 (50.0%)	2 (50.0%)
TSS1500	38	9 (23.7%)	6 (66.7%)	3 (33.3%)
5′ UTR	73	28 (38.4%)	11 (39.3%)	17 (60.7%)
Body	401	124 (30.9%)	70 (56.5%)	54 (43.5%)
Body	353	109 (30.9%)	62 (56.9%)	47 (43.1%)
3′ UTR	48	15 (31.3%)	8 (53.3%)	7 (46.7%)

Strongest negative correlations were observed for *ARL15*, *EPB41L2*, *ZNF516*, *WSB1*, *CDK6*, *TRPM1*, *RASSF8*, *AQP11*, *DENND2D* and *MAPK14* (Supplementary Table 3). Strongest positive correlations were observed for *ANTXR2*, *CTTN*, *CAMTA2*, *TMEM45A*, *SNX29*, *C1S*, *FYN*, *ANKRD55*, *KLF7* and *AKAP13* (Supplementary Table 3).

### Gene Ontology (GO) and pathway analyses

**Figure 5:**
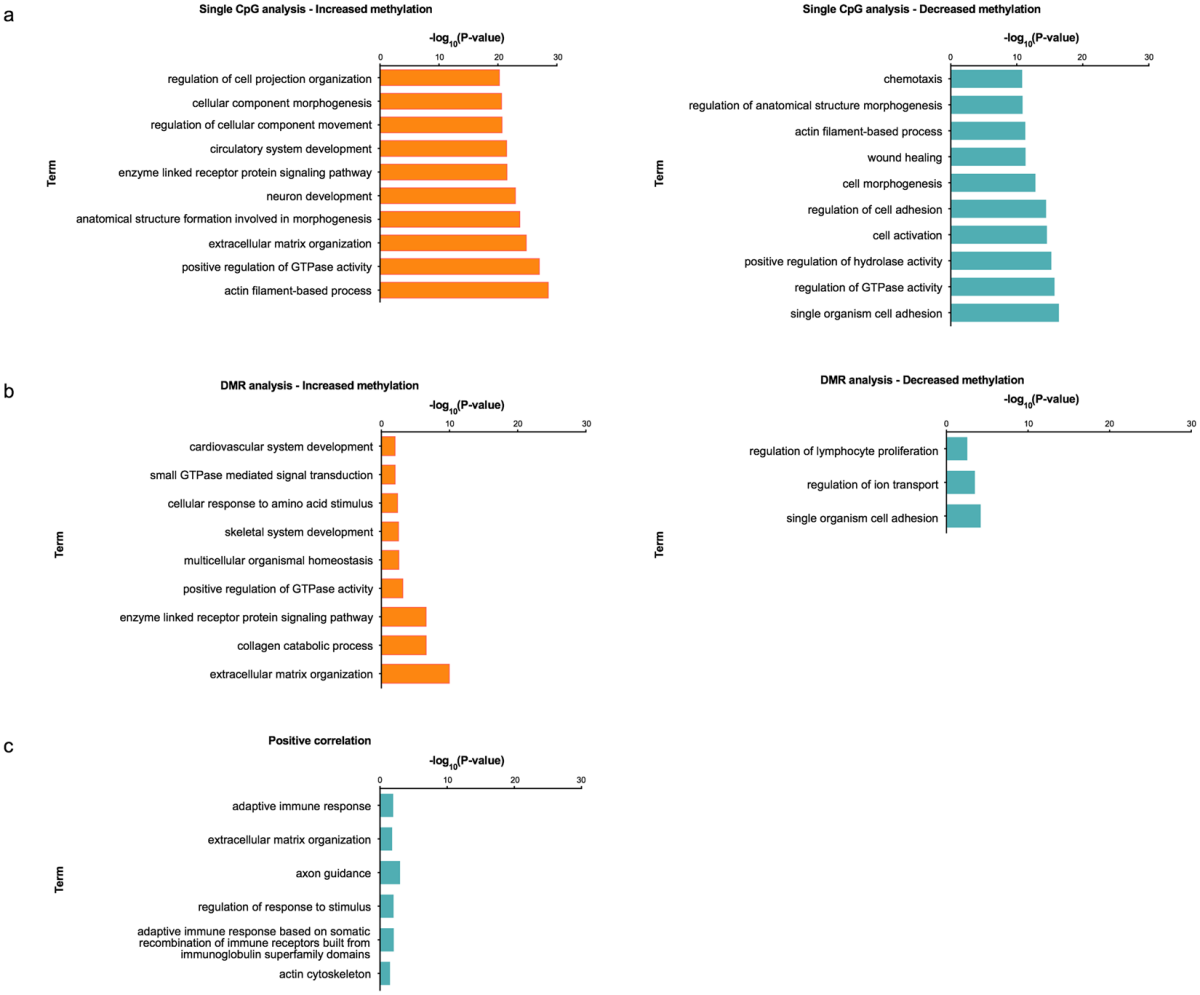
Pathway analysis of genes mapped to significantly differentially methylated sites. (**a**) CpG-level analyses. ‘Increased’ and ‘decreased’ methylation stand for methylation status in receptive endometrium relative to pre-receptive endometrium; (**b**) Region-level (DMR) analyses. ‘Increased’ and ‘decreased’ methylation stand for methylation status in receptive endometrium relative to pre-receptive endometrium; (**c**) For genes showing positive correlation between gene expression and methylation. No enrichment for biological terms was seen among negative correlations. The barplot shows the –log10 (p-values) for most significantly enriched pathways and GO terms. For full lists, please see Supplementary Tables 4–6).

In order to characterize the genes annotated to differentially methylated sites and regions, gene ontology and pathway analyses using g:Profiler^12^ and PANTHER^13, 14^ were carried out, and g:Profiler results were aggregated using GOsummaries^14^. In site-level analyses, we used the 22,272 differentially methylated CpGs, and the gene ontology analyses were performed separately for 1,464 and 5,196 genes associated with lower and higher methylation levels in receptive endometrium, respectively (according to CpG annotation). 681 genes were present in both categories, depending on CpG annotation. As shown in Fig. 5a, in site-level analyses, the genes affected by decreased methylation were mainly associated with immune response regulation and cell activation and adhesion, while genes associated with increased methylation were related to extracellular matrix organization, cellular signalling, regulation and development (Supplementary Table 4). This is largely mirrored by region-level analyses of DMRs, involving 1,206 genes associated with increased methylation and 275 with decreased methylation in receptive phase, respectively, which show that processes related to extracellular matrix and cellular adhesion are most affected by differential methylation (Fig. 5b, Supplementary Table 5). To functionally annotate the genes showing correlation between site-level methylation and gene expression (72 negative and 85 positive correlations), we used gene ontology analysis, which showed that positively correlated genes are related to extracellular matrix organization (*ITGAE*, *LAMA4*, *NID1*, *TGFB3*, *COL4A2*, *ADAMTS1*, *VCAM1*, and *COL6A2*) and immune response (*FYN*, *BCL3*, *PVR*, *JAK3*, *IL1RL1*, *RFTN1*, *MYO1G*, *CXCL13*, and *C1S*), while no enrichment in biological terms was seen for negative correlations (Fig. 5c, Supplementary Table 6).

PANTHER pathway analyses for the same gene lists showed enrichment in 16 pathways in site-level analysis, including VEGF signalling, oxytocin receptor mediated signalling, endothelin signalling, angiogenesis, integrin signalling, EGFR signalling, Wnt signalling, GnRH receptor and chemokine/cytokine signalling mediated inflammation pathways (for details see Supplementary Table 7). No enrichment was seen in region-level analysis; however, genes for which we observed correlation between methylation and gene expression were enriched for integrin signalling pathway genes.

## Discussion

The current paper describes the methylation landscape in pre-receptive and receptive endometrium of healthy fertile-aged women within one menstrual cycle, showing multiple small-scale changes that correlate well with changes in gene expression.

Previously it has been shown that the endometrial methylome is dynamic and changes throughout the menstrual cycle^7, 8^. However, these studies have compared different women with different menstrual cycle phases, thereby raising the question of how many of the described changes are due to true biological changes and not inter-individual variability^7, 8^. Furthermore, although the dynamic nature of endometrial methylome has been demonstrated, no study has used precisely timed tissue samples to investigate the methylation changes taking place at the time endometrial receptivity is established. Our study is the first to use precisely dated and histologically confirmed endometrial biopsies taken from the same women within the same menstrual cycle to eliminate inter-individual and inter-cycle variability. Such design targets the transition from pre-receptive to receptive phase of the endometrium to better characterize the potential methylation changes taking place during this limited period that could help to unravel the biological mechanisms responsible for endometrial receptivity. In our dataset, the comparison of methylation profiles showed no large-degree differences between early- and mid-secretory endometrium. However, we detected small-scale changes in methylation in a number of CpG sites. Since various methods use slightly different statistical approaches for detecting differential methylation, we used three methods and considered only those sites differentially methylated that were identified by all used methods. This way the methods are likely to complement each other and thus improve the reliability of our results.

Both site- and region-level analysis identified CpGs annotated to *ZMIZ1* as one of the top significantly differentially methylated genes. ZMIZ1 is a transcription factor regulator that among others regulates the androgen receptor, Smad3/4 and p53 signalling, the latter has also been associated with endometrial receptivity^15, 16^. Differentially methylated sites were also mapped to several genes with known function in endometrial receptivity and embryo implantation, including *PAEP*, *MAP3K5*, *ENPEP*, *GPX3*, *ARID5B*, *AOX1*, and *ANXA4* ^17^. Furthermore, ontology and pathway analyses of the genes annotated to differentially methylated sites/regions highlighted several pathways with established role in endometrial receptivity, such as immune response, Wnt signalling, angiogenesis and VEGF signalling, cell adhesion and extracellular matrix remodelling^18^.

Previous studies exploring the endometrial methylome have reported sites in or near *FAM181A*, *UXT*, *KRT34*, *KRTAP17-1*, *LASS3*, *CCL4*, *ST6GAL1*, *ZNF143*, *CYSLTR2*, *TDGF1*, *RANBP3L*, *SNORD109A*, *TRIM74*, *ACOT2*, *WT1*, *TCEAL4*, *MPP7*, *CASP8*, *PTPRN2* and *HCP5* as differentially methylated between the early- and mid-secretory phases^7, 8^. Our study confirmed the differential methylation of *KRTAP17-1*, *CASP8*, *RANBP3L*, *WT1*, *MPP7*, *PTPRN2*, and *HCP5*. Not much is known about the roles of *KRTAP17-1*, *RANBP3L*, *MPP7*, *HCP5* and *PTPRN2* in endometrial biology. However, *CASP8* has been shown to be among the genes dysregulated in women with chronic endometritis and impaired receptivity^19^, and IVF treatment failure^20^, while WT1 is associated with decidualization in rat endometrial stromal cells^21^, and is downregulated during WOI in polycystic ovary syndrome patients^22^. These lines of evidence support their role among the genes modifying the microenvironment within the receptive endometrium. Interestingly, *PTPRN2* was also among the genes that show a correlation between methylation and gene expression in our study, as two CpGs annotated to *PTPRN2* were negatively correlated with gene expression. Despite different study designs and relatively small overlaps, the aforementioned seven genes have been identified as differentially methylated between early- and mid-secretory endometrium in more than one study^7, 8^, proposing them as interesting candidates for further investigation.

We also correlated the differentially methylated CpGs with the greatest absolute changes in methylation levels with corresponding transcript levels and observed numerous correlations. There is no consensus on the extent of change in methylation needed to impact gene expression, as it probably depends on multiple additional regulatory factors and also on whether whole tissue or distinct cellular subpopulations are studied. However, small absolute changes in methylation have previously been found to associate with gene expression both on whole tissue^7^ and cell population^23^ level. Correlation analysis of methylation and gene expression levels revealed both positive and negative correlations in varying proportions depending on the genomic region. This is in accordance with recent studies showing that methylation can affect gene expression in both directions^24, 25^. However, as expected, we observed more negative correlations in the 5′ UTR while positive correlations were more common in the gene Body region. This is consistent with the ‘DNA methylation paradox’, whereby methylation of the transcribed region and region of transcription initiation have opposite effects on gene expression^11^. The proportion of negative and positive correlations is somewhat different from what Houshdaran *et al*.^7^ showed, as in their study, positive correlations were substantially more prevalent (70% positive vs 30% negative). Furthermore, the absolute number of observed correlations is also different between our study (169 correlations) and Houshdaran’s (40 correlations)^7^. However, it should be pointed out that the methodology used for methylation and gene expression profiling was different in our and Houshdaran’s study, and we used a paired study design, which could be the source for discordances and makes it difficult to compare the results.

Gene ontology and pathway analyses indicated that genes with a correlation between methylation and gene expression were related to extracellular matrix organization, integrin signalling and immune response, which are all important for endometrial function, and establishment of receptivity via tissue remodelling and modifying maternal immunity to facilitate implantation of the semi-allogenic embryo^18^. Genes related to extracellular matrix organization and immune response with positive correlation between methylation and expression levels included those that have previously been associated with endometrial receptivity, decidualization and embryo implantation either in humans or animal models, such as *TGFB3* ^26^, *ADAMTS1* ^27^, *VCAM1* ^28^, *IL1RL1* (also known as *ST2*)^29^, *CXCL13* ^30^ and *BCL3* ^31^. Interestingly, a direct link between *BCL3* methylation and expression has also been shown in mouse endometrial cells^31^. Although negative correlations were not enriched for any specific biological terms, they also involved genes linked with processes associated with endometrial receptivity, such as *CDK6* ^32^, *PTCH1* ^33^, *TDO2* ^34^, and *ETS2* ^35^. However, the observed statistical correlations need additional functional studies to determine the causal effect of methylation change on gene expression level.

### Strengths, limitations and future directions

The current study is the first using a study design targeting specifically the pre-receptive and receptive phases of the endometrium, and large-scale genome-wide approach to characterize the endometrial tissue methylome and its correlation with gene expression. By investigating samples from two time-points from the same women within the same cycle and evaluating methylation and gene expression within the same sample, we reduce inter-individual and inter-cycle variability and provide insight into the potential biological effects of methylation changes relevant for establishing endometrial receptivity and maintaining endometrial function. For methylome profiling we used the Illumina HumanMethylation450 array, one of the most comprehensive and high-resolution arrays for this purpose, while for obtaining gene expression data, we used RNA sequencing, which is more specific and sensitive, and with a broader dynamic range for quantifying gene expression levels compared to array technology. Due to the fact that several methods are available for differential methylation analysis, with no proper benchmark, we also used multiple analysis methods for detecting site-level differential methylation, enabling to select differentially methylated sites with higher confidence. In addition, since only site-level analysis ignores potential correlation between sites and can provide redundant results, we also evaluated region-level differential methylation, which offers improved statistical power^36^ and sensitivity^37^.

When interpreting the results of our study, it must be borne in mind that the sample size was rather limited (a total of 34 biopsies from 17 women for differential methylation analyses, and 14 biopsies from 7 women for methylation-gene expression correlation), which means replication in a larger dataset is required. Our study had 60% power to detect (at a nominal significance level of 0.05) CpG level absolute delta-$\beta$ changes equal to or larger than ~0.2.

Furthermore, we studied endometrial whole tissue biopsies that contain various cell types (stroma, epithelium, immune cells etc), each with potentially distinct methylation patterns, which are ‘diluted’ in whole tissue samples; therefore, methylation profiling of distinct endometrial cell populations separated by cell sorting or other methods is warranted and highly anticipated. If such a dataset becomes available for endometrial tissue or cells, it would also be interesting to consider the histone modifications around differentially methylated sites and regions to further understand the epigenetic regulation of gene expression in the endometrium.

## Conclusion

Our study offers insight into the methylation pattern and correlation between methylation and gene expression during pre-receptive and receptive phase in the human endometrium, showing that the overall methylome remains relatively stable during this stage of the menstrual cycle, with small-scale changes affecting only 5% of the studied sites. The generalized results of our analyses indicate that extracellular matrix organization and immune response are the most likely pathways regulated by methylation changes. Altogether, these results provide another piece of the puzzle for understanding the molecular mechanisms governing endometrial biology and receptivity and highlight the need for similar studies in distinct endometrial cell populations.

## Material and Methods

### Ethics statement

The study was approved by the Ethics Review Committee of the University of Tartu, Estonia (permission no 221/M-31). An informed consent was signed by all women before tissue collection and all methods were carried out in accordance with relevant guidelines and regulations.

### Patient characteristics

Endometrial biopsies (17 paired biopsies, a total of 34 biopsies) were obtained from 17 healthy fertile-aged volunteers (≤35 years; average ± standard deviation 30.1 ± 3.4) with average body mass index 23.6 ± 4.4. All women selected for the study reported regular menstrual cycles (25–35 days) and were clinically examined for the absence of hormonal dysbalance and/or uterine pathologies. The women self-reported to be non-smokers with no previous infertility records and had at least one live-born child. No participants had taken hormonal medications at least three months before entering the study. Endometrial tissue biopsy was obtained using Pipelle catheter (Laboratoire CCD, Paris, France) on day two and eight ( ± 1 day) after the LH surge (LH + 2 and LH + 8, respectively) within one natural cycle. These two time-points in the early- and mid-secretory endometrial phase correspond to the pre-receptive and receptive endometrium, respectively. Before taking the biopsy, the occurrence of ovulation was confirmed by ultrasound. LH surge was identified using commercial LH kits (BabyTime® hLH urine cassette, Pharmanova). Part of the collected endometrial tissue was stored in formaldehyde for histological confirmation of endometrial phase, while the rest was frozen at −80 °C in RNAlater (Ambion Inc., Austin, TX, USA). The endometrial phase (early secretory for pre-receptive time-point and mid-secretory for receptive time-point) was histologically confirmed for all biopsies included in this study.

### DNA extraction and DNA methylation measurement

Genomic DNA was isolated from approximately 20 mg of endometrial tissue using AllPrep DNA/RNA/miRNA Universal Kit (Qiagen, Venlo, The Netherlands) according to manufacturer’s original protocol. DNA hybridization to Infinium HumanMethylation 450 K BeadChip (Illumina, San Diego, CA, USA) was performed at USC Epigenome Center (Los Angeles, CA, USA) according to manufacturer’s specifications. Raw intensity files in IDAT format were used for all following analysis steps.

### RNA extraction and sequencing

For total RNA extraction, up to 30 mg of tissue was homogenized in the presence of QIAzol reagent (Qiagen) and processed using miRNeasy Mini kit (Qiagen), following manufacturer’s protocol. Purified RNA quality (all RIN > 7.5) was evaluated using Bioanalyzer (Agilent Technologies, Waldbronn, Germany). To perform transcriptome sequencing, cDNA libraries were generated from ~1 μg of endometrial total RNA using Illumina TruSeq technology (Illumina), following cDNA quality control with Bioanalyzer. RNA sequencing (RNA-seq) was performed at the Estonian Genome Center Core Facility using Illumina paired-end 100 bp sequencing technology according to manufacturer’s specifications. The sequenced data was trimmed and adapters removed with Trimmomatic-0.32^38^. Reads were quality filtered with FASTQ quality filter tool from FASTX-Toolkit v.0.0.14 and mapped with TopHat2^39^ on Human genome version 19. The transcript counts were extracted with HTSeq-count script^40^ from mapped data and further processed with Bioconductor package edgeR, which is designed for the analysis of count-based [count-per-million (CPM)] expression data^41^. The CPM values provided by edgeR were used for further correlation analysis and the CPM values for the transcripts used in correlation analyses (see below) are given in Supplementary Table 8. No additional filters for CPM values were used.

RNA-seq results were selectively confirmed by quantitative real-time PCR. Details of the differential expression analysis results, which are a part of a larger endometrial transcriptome dataset, will be presented in a separate paper (Suhorutshenko *et al*. in preparation).

### Normalization of methylation data

Data quality control and preprocessing were performed using the Bioconductor package RnBeads ver. 1.1.8^42^. The methylation β-value (ratio of methylated probe intensity over total intensity, ranging from 0 to 1) for each CpG probe was calculated according to Illumina’s formula β = m/(m + u + 100), where ‘m’ stands for methylated probe intensity and ‘u’ for unmethylated probe intensity. The methylumi-implemented Illumina scaling normalization was used, which fits with our data according to clustering (Supplementary Figure 1) [https://www.bioconductor.org/packages/release/bioc/html/methylumi.html]. Probes targeting the last 3 bases of sequence that overlaps with a single nucleotide polymorphism (SNP) were filtered out, as were cross-reactive probes^43^. In the first filtering step, 4,823 sites were removed because they overlap with SNPs, 30,378 probes were removed because their sequences were non-specific and have a high likelihood of cross-hybridization, and 1,703 probes were removed because the RnBeads ‘GreedyCut’ algorithm identified them as unreliable measurements across samples. In total, 36,904 probes were removed during initial filtering. In the second filtering step (includes the normalization procedure) a total of 11,651 probes were removed, 10,287 of which were located on sex chromosomes and the rest were context-specific non-CpG probes. At the end of filtering, 437,022 out of 485,577 probes remained for subsequent analysis.

### Genomic annotation of CpGs

The genomic regions for the CpG sites were annotated using the annotation file provided by Illumina. For the location relative to a gene, the following categories were used: TSS1500 (1,500 bp upstream from transcription start site – TSS), TSS200 (200 bp upstream from TSS), 1^st^ Exon, 5′ UTR (5′ untranslated region), Body (gene body), and 3′ UTR (3′ untranslated region). For the location relative to a CpG island (CGI), we used the following categories: island (CGI), S_Shore and N_Shore (up to 2 kb up- and downstream of the CGI), S_Shelf and N_Shelf (2–4 kb up- and downstream of the CGI), OpenSea (all others). When analysing the correlation between DNA methylation and gene expression, TSS1500, TSS200, 5′ UTR and first exon were grouped as the ‘5’ region’, whereas gene body and 3′ UTR were grouped into ‘gene body’. Due to alternative transcription start sites and several genes in one region, 327 (0.07%) CpGs in total and 13 (7.7%) sites among the significantly correlated ones were assigned multiple annotations. To test for differences in methylation value distributions between genomic regions, we carried out pairwise comparisons using the Kolmogorov-Smirnov test.

### Differential methylation analysis

For differential methylation analysis, three different approaches were used to increase the probability of achieving true positive results. Combining information from multiple methods can reduce the proportion of false positive findings and generalize the results with higher confidence, thereby increasing the reliability of the results. To make the results comparable and because the M-value is more statistically valid for differential methylation analyses^44^, all differential methylation analyses were conducted using M-values (defined as log2 ratio of methylated and unmethylated probe intensities) calculated with lumi R package^45^. All differential methylation analyses were adjusted for age due to the effect it has on methylation levels^46^.

For single CpG level differential methylation analysis, we used RnBeads^42^, seqlm^37^, and since we detected a slightly abnormal distribution in our data, also Wilcoxon signed-rank test. False discovery rate (FDR) adjusted p-value < 0.05 was considered as the statistical significance threshold. In the seqlm analysis, no limiting criteria were defined and all CpG sites with a FDR < 0.05 were extracted to make reasonable comparison with other methods. Eventually, the intersection between the three sets of significant differentially methylated CpGs generated by used programs was determined to define the most likely set of truly differentially methylated sites.

In addition to site-level analyses, we also performed region-level analysis using seqlm to detect differentially methylated regions (DMRs). In this analysis, DMR search criteria were the following: at least 3 consecutive differentially methylated CpGs (FDR < 0.05) within a 500 bp window.

To compare the distribution (in relation to gene subregions and CGIs) of differentially methylated CpG sites to the overall distribution of all analysed CpGs (n = 437,022) on the array, we used the $\chi$ ^2^ test.

### Correlation between methylation and gene expression

For the correlation analysis between RNA-seq and differential methylation analysis results, we used 7 individuals from the original dataset (n = 17) for whom both DNA methylation and RNA-seq data was available for both time-points (7 biopsies from pre-receptive and 7 biopsies from receptive time-point). Only those CpGs that were significantly differentially methylated in all three analyses with an absolute delta-β values > 0.1 were used for correlation analysis. CPM values provided by edgeR^41^ and significantly differentially methylated site β-values from RnBeads were used. We only evaluated *cis*-correlations, therefore we tested for correlations between a given CpG and the gene to which it was annotated. Spearman’s rank correlation coefficient was used to calculate correlations and the permutation p-values were used to evaluate the significance. For the significantly correlated CpG-gene pairs, if some region of gene of interest contained more than one CpG site, a median correlation value was calculated by region, e.g. 5′ UTR, 3′ UTR, Body, 1^st^ Exon, TSS200 and TSS1500. For example, CpGs in the *C1QTNF7* gene were located in 3 different regions (TSS200, Body and 5′ UTR). One CpG is located in the 5′ UTR, while Body and TSS200 regions contained two sites each. The median correlation was calculated separately for the *C1QTNF7* TSS200 and *C1QTNF7* Body.

### Gene ontology (GO) and pathway analyses

GO enrichment analysis was performed for the genes mapped to significantly differentially methylated CpGs and DMRs, and for the differentially methylated genes with significant correlation with gene expression using the web tools g:Profiler^12^ and PANTHER (v11.1)^13^. For graphic representation of the g:Profiler analyses we aggregated the results using the Bioconductor package GOsummaries^14^ which internally uses g:Profiler with numerous filtering criteria in order to achieve non-redundant summaries. p-values from the g:Profiler analysis (corrected for multiple testing using the g:SCS algorithm implemented in g:profiler)^12^ were used for depicting the pathway analysis results.

### Accession codes

All of the Illumina HumanMethylation450 DNA methylation data are available at the GEO database (accession number GSE90060).

## Electronic supplementary material

Supplementary Figures

Supplementary Dataset 1
