# Supplementary material for: DNA methylation changes in endometrium and correlation with gene expression during the transition from pre-receptive to receptive phase
Source: Sci Rep. 2017 Jun 20;7:3916. doi: 10.1038/s41598-017-03682-0 (PMC5478666; doi:10.1038/s41598-017-03682-0)
Supplement: Supplementary file 1 — Supplementary Figures [file 41598_2017_3682_MOESM1_ESM.docx]

**Supplementary figures**

**Manuscript title:** DNA methylation changes in endometrium and correlation with gene expression during the transition from pre-receptive to receptive phase

Viktorija Kukushkina & Vijayachitra Modhukur, Marina Suhorutšenko, Maire Peters, Reedik Mägi, Nilufer Rahmioglu, Agne Velthut-Meikas, Signe Altmäe, Francisco J. Esteban, Jaak Vilo, Krina Zondervan, Andres Salumets, Triin Laisk-Podar

Supplementary figure 1. Clustering of analyses samples after methylumi-implemented Illumina scaling normalization. ES – early-secretory (pre-receptive LH+2) samples; MS – mid-secretory (receptive LH+8) samples.


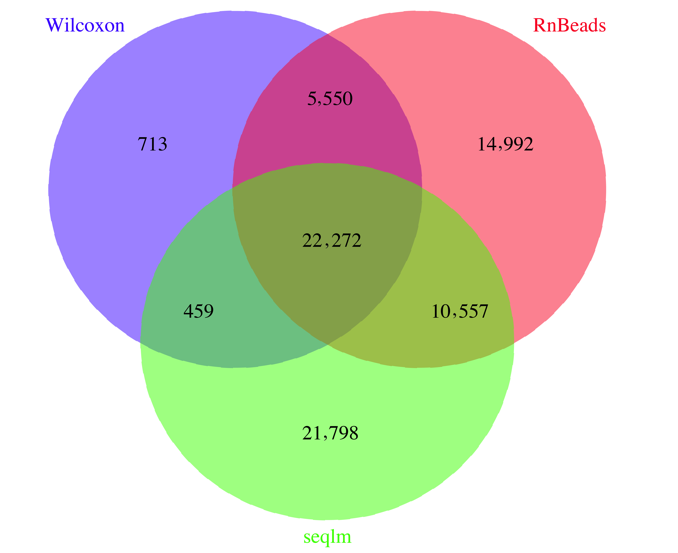


Supplementary figure 2. A Venn diagram depicting the overlap between the three methods used to detect differentially methylated CpG sites between pre-receptive and receptive phases of endometrium from the same individuals (n=17).

Supplementary figure 3. Clustering of samples according to the 22,272 differentially methylated CpGs. ES – early-secretory (pre-receptive LH+2) samples; MS – mid-secretory (receptive LH+8) samples.
